# Usage of Converter Gas as a Substitute Fuel for a Tunnel Furnace in Steelworks

**DOI:** 10.3390/ma15145054

**Published:** 2022-07-20

**Authors:** Dorota Musial, Magdalena Szwaja, Marek Kurtyka, Stanislaw Szwaja

**Affiliations:** 1 Faculty of Production Engineering and Materials Technology, Czestochowa University of Technology, Armii Krajowej Avenue 19, 42-200 Czestochowa, Poland; dorota.musial@pcz.pl; 2 Department of Thermal Machinery, Faculty of Mechanical Engineering and Computer Science, Czestochowa University of Technology, Armii Krajowej 21, 42-200 Czestochowa, Poland; magdaszw24@gmail.com; 3 Termo-Klima MK, Tartaczna 12, 40-749 Katowice, Poland; marekkurtyka@onet.pl

**Keywords:** converter gas (BOFG), steel processing, ecological and economic benefits, skeletal mechanism, DRGEPSA method

## Abstract

Converter gas (BOFG) is a by-product of the steel manufacturing process in steelworks. Its usage as a substitute fuel instead of natural gas for fueling a metallurgical furnace seems to be reasonable due to potential benefits as follows: CO_2_ emission reduction into the ambient air and savings in purchasing costs of natural gas. Results of theoretical analysis focused on implementing converter gas as a fuel for feeding a tunnel furnace for either steel plate rolling, steel sheet hardening in its real working condition or both, are discussed. The analysis was focused on the combustion chemistry of the converter gas and its potential ecological and economic benefits obtained from converter gas usage to heat up steel in a tunnel furnace. Simulations of combustion were conducted using a skeletal chemical kinetic mechanism by Konnov. The directed relation graph with error propagation aided sensitivity analysis (DRGEPSA) method was used to obtain this skeletal kinetic mechanism. Finally, the model was validated on a real tunnel furnace fueled by natural gas. Regarding exhaust emissions, it was found that nitric oxide (NO) dropped down from 275 to 80 ppm when natural gas was replaced by converter gas. However, carbon dioxide emissions increased more than three times in this case, but there is no possibility of eliminating carbon dioxide from steel manufacturing processes at all. Economic analysis showed savings of 44% in fuel purchase costs when natural gas was replaced by converter gas. Summing up, the potential benefits resulting from substituting natural gas with converter gas led to the conclusion that converter gas is strongly recommended as fuel for a tunnel furnace in the steel manufacturing process. Practical application requires testing gas burners in terms of their efficiency, which should provide the same amount of energy supplied to the furnace when fed with converter gas.

## 1. Introduction

The iron and steel industry are sectors characterized by high energy consumption and significant air pollution. It is estimated that around 5–10% of global energy is consumed by the steel industry, from which carbon dioxide emissions account for about 4–7% of total anthropogenic CO_2_ emissions [1,2]. According to statistical data [3], the steel industry emits approximately 9.8% of worldwide particulate pollutants. CO_2_ emissions (98%) constitute the largest share in the form of gaseous pollutants emitted in the steel industry. Emissions of other gases: NO_2_, SO_2_, and CO, constitute 2%. Industrial combustible gases formed in steelworks are inconvenient to manage. However, this waste can be treated as a fuel characterized by relatively high energy content. Thus, the energy usage of waste industrial gases should be one of the priorities of the steel industry, and its task is to improve both the ecological and economic efficiency of steel manufacturing technologies. One of the easiest ways to manage gases is to burn or co-burn them directly at the place of their origin. However, it is difficult to maintain the required gas purity and continuity of gas supply with appropriate energy parameters. In this article, converter gas (BOFG) is proposed to be used as a substitute fuel instead of natural gas for fueling a metallurgical furnace.

As two main themes are important, the introduction section has been divided into two main parts focusing on the following:

- justification of converter gas to be used as a substitute fuel in the steel manufacturing process,
- tools for modeling and chemistry reduction methods, which can be implemented for converter gas modeling with the aid of a skeletal combustion mechanism.

One of the three main gases produced in the integrated steel production process is basic-oxygen-furnace gas (BOFG or converter gas). BOFG is a by-product generated in the oxidation of pig iron in the oxygen converter, and its yield depends on the method of gas recovery used and its use as an energy source. Using an “open combustion system”, 2000–3000 m^3^ of BOFG per tonne of liquid steel is formed. However, only 50–100 m^3^ of BOFG per tonne of liquid steel is formed in “suppressed combustion” systems. As Zhai et al. stated [4], converter gas (BOFG) composition varies depending on the following: the process used, the recovery method, and the volume of oxygen. Crude BOFG contains approximately 56–70% CO, 13–20% N_2_, 15–21% CO_2_, and small amounts of 1–4% H_2_. It is a low calorific gas with a calorific value in the range between 8.0 and 9.0 MJ/Nm^3^. As a low calorific gas, it features a relatively slow combustion rate. This observation was additionally confirmed by others [5,6,7]. Hence, the main target and the essence of this article is to perform an analysis of applying BOFG as a fuel for a tunnel furnace that heats up steel sheets either for a hot rolling mill, steel hardening, or both. Thus, the content is divided into three sections:

- testing and verification of a skeletal reduced kinetic combustion mechanism obtained with the aid of the DRGEP method,
- modeling combustion of BOFG in a tunnel furnace with aid of this tested and verified mechanism with a focus on ecological aspects,
- economic benefits from applying the BOFG to the tunnel furnace.

As known, several countries and regions established targets for CO_2_ reduction in order to meet the sustainability goals agreed upon under the Kyoto Protocol [8]. Therefore, steel manufacturing processes are forced to modernize production processes in order to meet their high ecological and economic requirements. The search for new methods to reduce CO_2_ emissions at all stages of the technology chain becomes particularly important. As Carpenter noticed [9], this action applies primarily to steel mills with a full production cycle, including coking plants, sinter plants, blast furnaces, and oxygen converters. Energy consumption in steel mills is limited by the following: introducing energy-saving devices into steel production processes, improving the efficiency of energy conversion plants, and the energy use of process gases, as stated by Biermann et al. [10] and Mittal et al. [11].

All gases generated in integrated smelters (coke oven gas—COG, blast furnace gas—BFG, and basic oxygen furnace gas, converter gas—BOFG) undergo a purification process. Among others, process gases after cleaning can be used by the smelters in the production cycle as follows:

- fire coke batteries in a coking plant (COG) [12];
- generate heat, steam, and electricity for the smelter’s own needs (COG, BFG) [13,14].;
- ignite the sinter mixture on sinter strands in ignition furnaces (a mixture of BFG and COG) [15];
- increase coke savings in blast furnaces (COG, BFG, BOFG, or COREX gas as hot reduction gas after removal (CO_2_)) [16,17];
- fire blast heaters (BFG with COG or oxygen-enriched blast with recirculation of blast furnace exhaust) [18];
- supply heating furnaces in hot rolling mills (COG, BFG) [19,20].

Unfortunately, in several smelters, excess process gases have been burnt in torches. This primarily affects the emission of unwanted pollutants into the atmosphere adversely. In addition, the smelter incurs economic losses as a result of not using raw materials with significant energy parameters. As Matino [21] discussed, production of blast furnace gas requires continuous optimal planning of the blast furnace gas usage according to its availability and to the needs in the steelworks. In connection with the above, it is reasonable to search for new solutions ensuring effective use of process gases directly at their place of formation. One such technology is the possibility of energetic combustion of BOFG in a heating furnace in a rolling mill.

Conducting experimental research, especially in natural industrial conditions, is often a difficult and time-consuming process. Under these circumstances, computational techniques become a remedy that is a more precise and practical solution in use. They make it possible not only to verify correctness of assumptions but also to perform analyses for conditions unattainable in experimental research and in the initial diagnostics of newly designed objects. In addition, it turns out that compared to experimental research, computational techniques are usually a much cheaper solution, because they do not require the use of complicated and expensive measuring equipment.

Available sources of literature contain various methods and numerical models, according to the authors, that correctly present the optimization of heating furnaces. Among others, Rimar et al. [22] proposed an analytical model for volumetric combustion with uniform temperature distribution for analyzing heat and flow processes in a metallurgical furnace. Furthermore, Yan et al. [23] implemented multi-computational fluid dynamics (CFD) ANSYS-Fluent software for modeling supersonic oxygen jets to model furnace gas combustion. They found that temperature, flow velocity, and turbulence intensity were reliably modeled. As a result of improving computational models and introducing new chemical reaction kinetics into mechanisms, an increase in the accuracy of calculations is observed as stated by Musial [24], Zajemska et al. [25], and Yu et al. [26]. The calculations use the entire available base of chemical models—from the simplest (containing several reactions) to the detailed (containing several hundred reactions). The choice of the appropriate mechanism is controversial. Selecting mechanisms should bear in mind that:

- the advantage of detailed mechanisms is the high accuracy and detail of the results obtained, and the disadvantage is the extended calculation time and associated procedure costs;
- the advantage of simplified mechanisms is the short calculation time, and the disadvantage is the lower accuracy of the results obtained;
- advantages of skeletal mechanisms include: the same accuracy of results as for detailed models and much shorter computation time.

Considering the advantages of using reduction methods, a large interest in skeletal mechanisms is observed. It is expected that reduction methods will become commonly used tools in creating simplified mechanisms for gaseous fuel combustion. Sarathy et al. [27] worked out a reduced chemical kinetic mechanism for singly methylated iso-alkanes ranging from C7 to C20 and found very good accuracy with experimental data. Furthermore, on the basis of the GRI Mech 3.0 mechanism, Hu et al. [28] worked out a minimal skeletal mechanism involving 20 species and 56 reactions for CH_4_/O_2_/CO_2_ mixtures. They used two methods as follows: the directed relation graph-aided sensitivity analysis (DRGASA) and the principal component analysis (PCA) for mechanism reduction. Finally, they validated this mechanism by several experiments and found that the developed skeletal mechanism could reproduce the results from the detailed mechanism. The DRGASA method was also confirmed as a successful tool for mechanism reduction by Liu et al. [29]. Skeletal mechanisms developed using the reduction method should only be used for those applications and boundary conditions for which they were created, as discussed by Liu et al. [29] and Zhao et al. [30]. However, it often happens that a skeletal mechanism developed on the basis of simple applications can be used for more complex simulations and still has satisfactory accuracy. In such cases, it is justified to use skeletal mechanisms developed for homogeneous reactors, i.e., PSR, subject to validation to confirm that the calculation procedure actually leads to the expected results. In fact, this is often the only way to obtain comprehensive reduced skeletal mechanisms from very large detailed mechanisms for calculating fuel combustion processes, especially for large industrial facilities. Among the many methods of reducing chemical mechanisms, these above-mentioned methods are some of the most frequently used. Sensitivity analysis is the final stage of the DRGEP method, which provides a relatively quick calculation time and a moderate reduction rate compared to other methods. Chen et al. [31] described the usefulness of the DRGEP method with the Dijkstra’s algorithm and the AStar algorithm for generating the reduced n-heptane mechanism with similar final results concerning laminar flames as the detailed mechanism. Niemeyer et al. [32] presented the DRGEP method for reducing the n-alkanes kinetic mechanism covering n-octane to n-hexadecane with 2115 species and 8157 reactions. They achieved the comprehensive skeletal mechanism consisting of 202 species and 846 reactions and the high-temperature mechanism consisting of 51 species and 256 reactions. Both mechanisms were validated and the authors found good correlation with results from the detailed mechanism in a perfectly-stirred reactor and laminar flame simulations. As Qiu et al. [33] pointed out, reduction of detailed mechanisms is of significant importance to CFD simulations. They obtained 10% error tolerance in calculations with their comprehensive reduced mechanism that was generated with 75% reduction of species and 80% reduction of reactions. Li et al. [34] investigated the pathways of the iso-octane mechanism and they found that the most important 10 reactions contribute almost 75% to the overall variance in ignition delay. The reactions are in small-molecule chemistry (C0–C4) and contribute significantly to uncertainties, mostly in the ignition predictions. In this article, the calculation procedure for the combustion of gaseous fuels was carried out using the detailed chemical mechanism, Konnov 0.6, which includes 129 compounds and 1231 chemical reactions [35]. As the method of reducing the mechanism, the following methods were chosen: directed relation graph with error propagation (DRGEP) and sensitivity analysis (DRGEPSA).

Summing up, the available literature resources do not contain results on practical implementation of BOFG gas into a tunnel furnace with the aid of modeling the combustion process with skeletal kinetic mechanisms and the DRGASA method.

## 2. Materials and Methods

An industrial system for heating up steel sheets consisted of a tunnel-heating furnace that was used for calculations. This furnace is located in one of the steel mills in Poland, however, the obtained results and conclusions can be transferred to any other steel mills working on the same principle all over the world. Considering that the smelter has surplus process gases, the main assumption was to determine the optimal solution in ecological and economic aspects, for using BOFG gas in a tunnel furnace.

### 2.1. Experimental Setup

**Figure 1:**
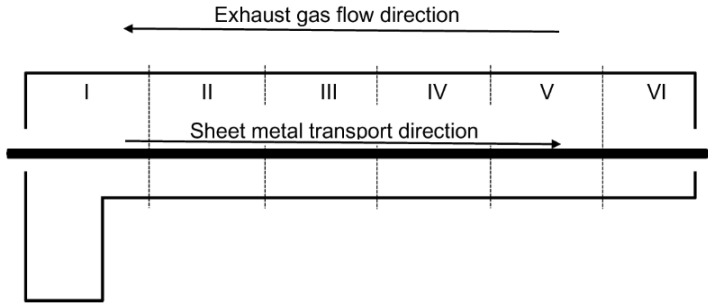
Construction scheme of the tunnel furnace with combustion zones I–VI.

The tunnel furnace for sheet hardening selected for testing is currently powered by natural gas (NG) supplied directly from a pressure-reduction station at overpressure of 10 kPa. NG, during the experiment, had the composition of: 96% CH_4_, 2% C_2_H_6_, 1.8% N_2_, 0.2% CO_2_. The internal dimensions of the furnace are: 49.5 m × 3.8 m × 2.34 m (length × width × height). The furnace is equipped with 133 burners installed on the sidewalls, regulating the operation of six control zones (Figure 1). The heated sheets move in a countercurrent direction in relation to the furnace exhaust gas flow.

During the experiment, the furnace worked at a yield of 9.5 Mg/h steel sheets, with a power of 7 MWth. Zones I–IV were maintained on a small excess of combustion air (*λ* = 1.05, equivalence ratio ϕ = 0.95), while zones V–VI did so on insufficient combustion air (*λ* = 0.90, equivalence ratio ϕ = 1.11) (Table 1). The composition of the exhaust gas at the gas outlet was measured with the Vario Plus-MRU exhaust gas analyzer and the analysis showed: 9% CO_2_, 1.5% O_2_, 15 ppmv CO, and 280 ppmv NO.

### 2.2. Calculation Procedure

The reduction of the Konnov 0.6 mechanism was carried out for a perfectly stirred reactor (PSR) at parameters as follows:

- pressure of 1 atm;
- initial temperature of 298 K;
- equivalence ratio of 0.7–1.1;
- residence time of 0.001–100 s.

The reduction of mechanisms was controlled by a normalized maximum error $\varepsilon_{EP}$ for three defined concentrations of emitted compounds: CO, NO, and NO_2_, setting criteria for which predictions of compound concentrations using RKM (reduced kinetic mechanism) should be consistent with predictions determined using DKM (detailed kinetic mechanism). It should be noted that with the change of the threshold value $\varepsilon_{EP}$, a skeletal mechanism with different levels of accuracy can be obtained. Herein, the smaller the $\varepsilon_{EP}$ value, the larger the skeletal mechanism that will coincide with the detailed mechanism when $\varepsilon_{EP}\;$approaches zero. If the maximum error of the next iteration of the skeletal mechanism exceeds the user-defined error limit, the threshold of the achieved goal is reduced. However, if the maximum error is below the error limit, the threshold is increased until the error reaches the specified limit. In order to generate the smallest possible skeletal mechanism by eliminating all irrelevant compounds omitted by DRGEP, with relatively low calculation costs, additional compound sensitivity analysis (DRGEPSA) was used. With the help of this method, insignificant compounds generated by DRGEP are sorted in ascending order based on the compound elimination error, which is the difference between the error caused by the elimination of the relationship in relation to the detailed mechanism and the error of the mechanism generated by DRGEP, and then removed from the mechanism. Finally, the minimum skeletal mechanism RKM_Konnov 0.6 was generated containing 42 compounds and 384 reactions.

### 2.3. Testing and Verification of the RKM_Konnov 0.6 Mechanism

**Figure 2:**
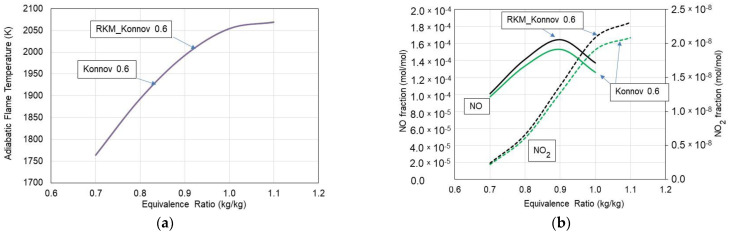
Adiabatic flame temperature (**a**) and NO, NO_2_ fractions (**b**) vs. equivalence ratio for Konnov and RKM_Konnov mechanisms.

Calculations were conducted with two kinetic mechanisms as follows: Konnov 0.6 and reduced RKM_Konnov 0.6 mechanisms. Figure 2a presents the adiabatic flame temperature vs. the equivalence ratio. As can be seen, there are no significant differences in both the calculated combustion temperature, NO, and NO_2_ exhaust emissions (Figure 2b) fractions for these mechanisms. The developed skeleton mechanism predicts these parameters with similar accuracies for the entire range of equivalence ratios under consideration. As observed in Figure 2b, higher numbers for NO and NO_2_ resulting from the RKM_Konnov 0.6 mechanism in comparison with the detailed Konnov 0.6 mechanism can be considered marginal, as long as the differences do not exceed 10% following recommendations by Niemeyer et al. [32] and Qiu et al. [33].

**Figure 3:**
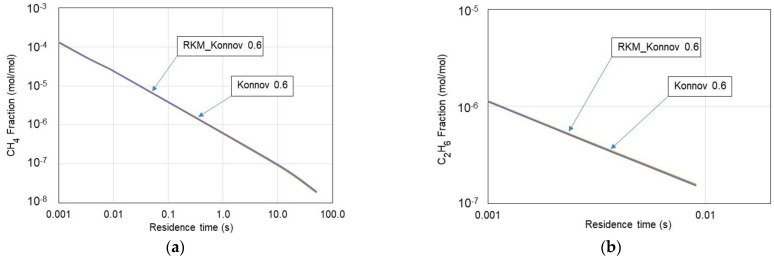
CH_4_ (**a**) and C_2_H_6_ (**b**) volume fractions vs. residence time in the PSR.

Furthermore, to verify the usability of the RKM_Konnov 0.6 mechanism, the time-based histories of CH_4_ and C_2_H_6_ fractions burnt were determined. Figure 3 presents the history of these gases in the PSR. As seen, no significant differences in the calculated volume fractions of CH_4_ and C_2_H_6_ were observed while applying these mechanisms to perform a comparison. The skeletal RKM_Konnov 0.6 mechanism accurately predicts volume distribution profiles over the entire range under consideration. Hence, further considerations were based on the RKM_Konnov 0.6 mechanism.

## 3. Results and Discussion

Results from the analysis and discussion presented in this section deal with ecological aspects concerning exhaust emissions from applying BOFG instead of NG as fuel to the tunnel furnace. Additionally, an economic analysis was conducted to show the benefits of applying BOFG as a substitute for NG for heating a tunnel furnace.

### 3.1. Ecological Aspect of Use of BOFG

Calculations of gas combustion in the tunnel furnace were carried out by treating the furnace as a reactor cluster. Each PSR reactor was assigned to a particular furnace zone in order to accurately reproduce the processes occurring there. As mentioned, the skeletal RKM_Konnov 0.6 mechanism was used in the calculations. The calculations adopted some simplifications while maintaining the real dimensions of the furnace and technical parameters, namely:

- constant furnace power P = 7 MWth;
- BOFG composition: 3% H_2_, 64% CO, 15% CO_2_, and 18% N_2_;
- flue gas residence time in individual zones (Table 2), calculated from furnace geometry and flue gas flow rate [36].

**Figure 4:**
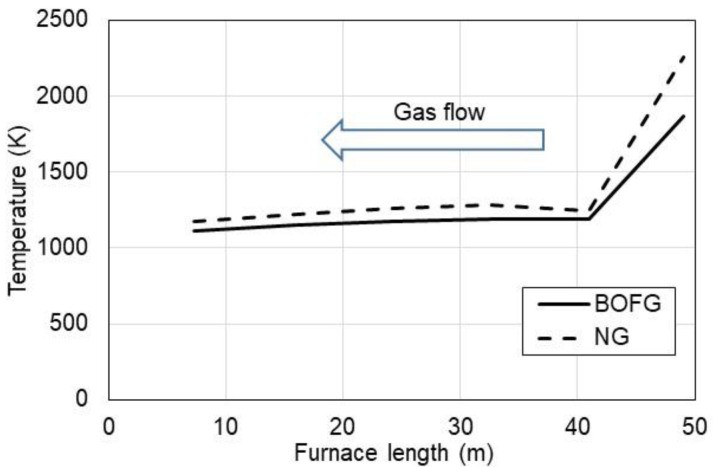
Exhaust gases temperature inside the tunnel furnace vs. length of this furnace fueled optionally with NG and BOFG.

Figure 4 presents the temperature of the flue gases calculated under boundary conditions for the steel sheet temperature and heat balance in the furnace based on the data provided in Table 2. As can be seen, the temperature of the flue gases rapidly decreases at the gas inlet (right side) due to the cooling effect by moving the metal sheet in the opposite direction to the flue gases as shown in Figure 1. In the next zones, the temperature of flue gases is around 100–150 K higher than the temperature of the steel sheet.

**Figure 5:**
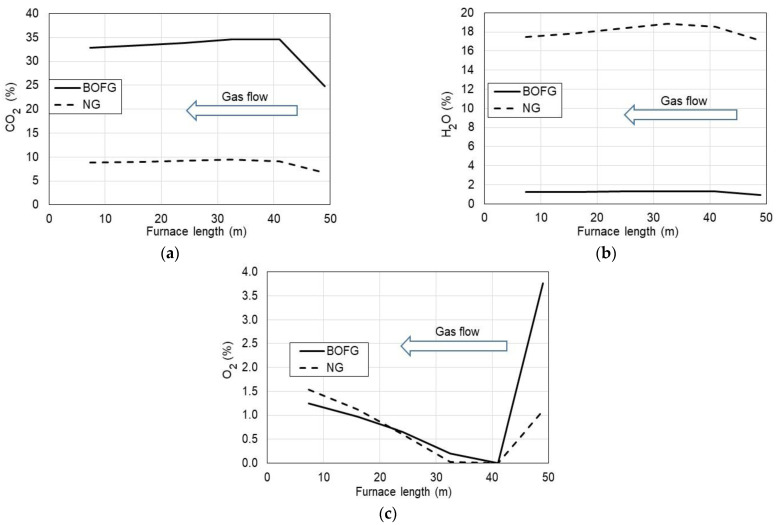
Combustion products CO_2_ (**a**), H_2_O (**b**), and O_2_ excess (**c**) volume fractions vs. length of the tunnel furnace fueled with NG and BOFG.

**Figure 6:**
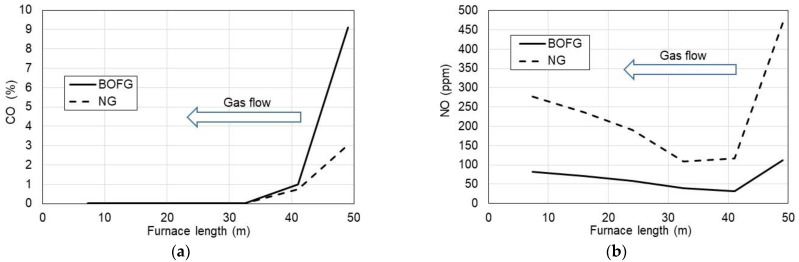
CO (**a**) and NO (**b**) fractions in the flue gases vs. length of the tunnel furnace fueled with NG and BOFG.

The calculated volume fractions of exhaust components (CO_2_, H_2_O, O_2_, CO, and NO) along the tunnel furnace length are shown in Figure 5 and Figure 6. BOFG gas shows a significantly higher fraction of CO_2_ in the combustion gases as compared to NG. The BOFG at the outlet of the furnace contained nearly 33% CO_2_, which is more than three times higher than CO_2_ in exhaust gases from NG. This CO_2_ content for BOFG combustion comes from the carbon balance in the BOFG fuel. As known, BOFG contains CO and CO_2_ content of 79% by volume and another combustible gas is H_2_ in the amount of only 3%.

As hydrogen in BOFG is present at the concentration of 3%, H_2_O in the product site is also expected to be several times lower in comparison with NG combusted (Figure 5b).

Regarding oxygen in the flue gases (Figure 5c), BOFG and NG are characterized with similar O_2_ content in the flue gases as a result of similar excess air provided to the combustion process.

A relatively high level of CO (Figure 6a) for both considered gases was observed at the gas inlet of the furnace (3.0% for NG and 9.1% for BOFG). However, at the furnace gas outlet, CO was reduced to a few ppm due to afterburning that resulted from oxygen presence in those zones due to an increase in excess air ratio λ.

As regards NO emission (Figure 6b), the highest NO fractions were observed for NG combustion at the gas inlet. It was caused due to the highest temperature which promotes NO formation following the thermal NO mechanism by Zeldovich. However, at the furnace gas outlet, NO concentrations were stabilized at the level of 275 ppm for NG and 80 ppm for BOFG. These values can be accepted with respect to the ecological point of view. On the other hand, NO emission from BOFG combustion was three times lower than that from the NG-fueled furnace. It was probably caused by higher amounts of inert gases (15% CO_2_ and 18% N_2_) which both work as diluents and slow down reaction rates. Thus, from the ecological point of view, BOFG as fuel to the tunnel furnace can be recommended due to lower NO emissions.

### 3.2. Economic Aspect of the Use of BOFG

Steel mills, in order to meet their high requirements, take all possible measures to improve the economic efficiency of their operations. The ideal assumption is to reduce the consumption of natural gas for the heating needs of the furnace. Natural gas can be co-incinerated with process gases generated in the smelter, or it can be eliminated by full substitution with these gases. Accordingly, an analysis was carried out, in which the two main goals were taken into consideration: comparison of BOFG consumption with natural gas in a heating furnace, and estimation of gas use costs. The amount of BOFG consumption was estimated using the so-called conversion factor for the change in the volume of natural gas to BOFG. It was calculated based on the relationship presented with Equation (1):

(1) $$\kappa_{x}=\frac{LHV_{NG}}{LHV_{BOFG}}$$

where: $LHV_{NG}$—lower heating value of NG $\left(35.74\frac{MJ}{Nm^{3}}\right)$ and $LHV_{BOFG}$—lower heating value of BOFG $\left(8.39\frac{MJ}{Nm^{3}}\right)$.

**Figure 7:**
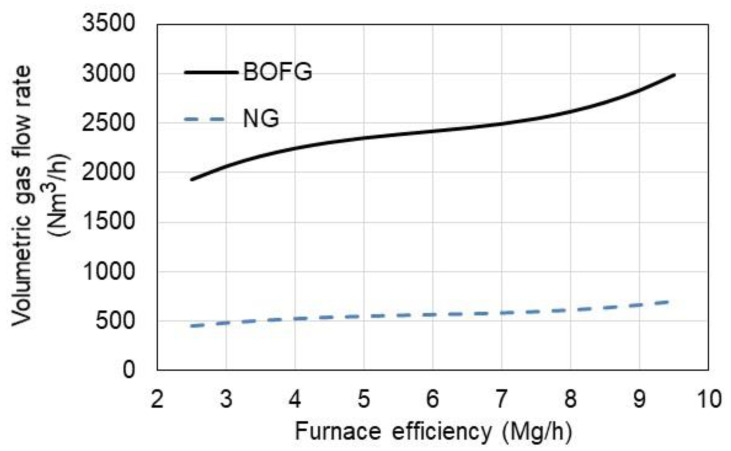
Gas consumption vs. furnace efficiency.

The calculation results are shown in Figure 7, depending on the furnace efficiency.

Due to the fact that BOFG has a much lower calorific value than NG, the volumetric consumption of BOFG is more than four times higher than in the case of NG. For the highest furnace efficiency of 9.5 t/h, BOFG consumption is observed at 2992 Nm^3^/h.

The purchase price of each gaseous fuel was estimated on the basis of data obtained from steel mills, assuming average values, respectively: NG—1.2 EUR/Nm^3^ (March–April, 2022) and BOFG—0.21 EUR/Nm^3^ (December 2021). As the price of NG has recently not been stable and the BOFG price is regulated by local demands, hence, the relation between BOFG and NG prices was initially expressed in arbitrary units (a.u.) assuming that 1 a.u. equals 1 EURO.

**Figure 8:**
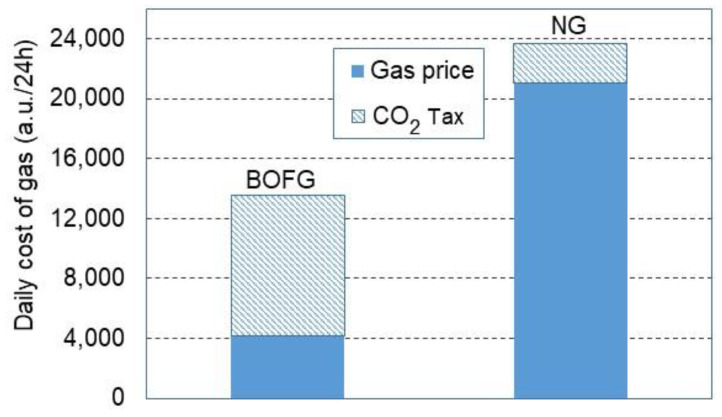
Daily cost of purchasing NG and BOFG including a carbon tax, net price, and total price.

It was also assumed that the total cost of purchasing the gaseous fuel for heating the tunnel furnace consists of the gas net price and the cost of the carbon tax. The carbon tax was assumed at 80 a.u./Mg CO_2_. The prices of the fuels were assumed as their specific prices referring to 1 MWh of energy stored in the fuel. Therefore, the net prices were set as follows: NG price of 125 a.u./MWh, whereas BOFG price of 25 a.u./MWh. As depicted in Figure 8, the daily overall purchase cost of BOFG is lower by approximately 44% than the NG total price, although, net prices differ much more from each other. As seen at prices of 25 and 125 a.u./MWh for BOFG and NG, respectively, the percentage ratio expressing BOFG price to NG price is 20%. Thus, the potential economic savings were estimated at the same percentage ratio. As depicted in Figure 8, the daily cost of BOFG is nearly 13,500 a.u./24 h, whereas purchasing cost of NG is 23,600 a.u./24 h. Thus, the savings promoting BOFG are at the amount of 10,100 a.u./24 h. Unfortunately, the BOFG cost is mainly charged with the carbon tax. On the other hand, this carbon tax has to be paid, even though the BOFG would not be used as a fuel for this furnace. Then, BOFG would be burnt in a torch, thus, CO_2_ would be emitted into the ambient air anyway. Additionally, one can conclude that by applying BOFG, CO_2_ reduction is evident due to eliminating natural gas from the process. Hence, CO_2_ reduction is exactly associated with NG removal from the steel manufacturing process at this stage. Thus, having this issue in mind, BOFG as fuel for heating the steel sheets in the tunnel furnace should be strongly recommended and implemented into practice if possible in any steel works.

**Figure 9:**
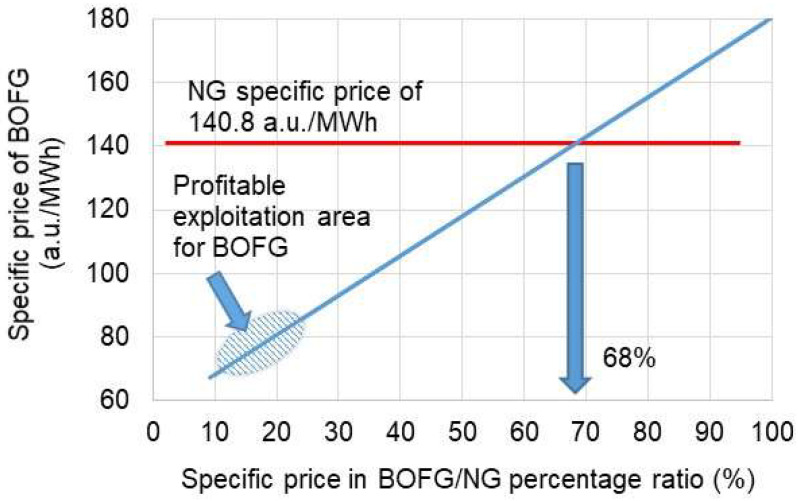
Specific prices for BOFG and NG vs. percentage ratio between BOFG and NG prices.

The potential savings for a factory can be generated on the basis of the cheaper cost of BOFG in comparison to NG. As shown in Figure 9, potential economic benefits can be generated in a company, which would like to introduce BOFG as fuel only under the condition that the BOFG specific price (a.u./MWh) ranges between 10 and 25% of the specific price of NG. In these circumstances, the real specific price of BOFG can be in the approximate range from 67 to 86 a.u./MWh. Otherwise, in the case of higher prices for BOFG, one can conclude that expected economic profits might be doubtful. However, the best scenario for implementing BOFG as a fuel can be achieved if BOFG is free of charge.

However, in the economic analysis, one cannot ignore an issue as important as the emission of greenhouse gases and the carbon tax for the emission of CO_2_ into the atmosphere. As shown (Figure 5a), the CO_2_ emission during the furnace treatment of BOFG is more three times higher than in the case of applying natural gas. On the other hand, BOFG as a by-product of steel manufacturing cannot be directly released into ambient air due to the toxicity of CO. Therefore, it has to be combusted, thus, it is already charged with the carbon tax.

## 4. Conclusions

Conducting experimental research on industrial facilities is a time-consuming process and often difficult to implement due to the inability to introduce measuring devices into the furnace workspace. For this reason, computational techniques are most often used. These are more precise and practical solutions and allow analysis to be carried out for conditions that are often unattainable in experimental research.

The following conclusions were drawn from the analysis:

- Combustion kinetic mechanism RKM_Konnov 0.6 was found as a reliable tool for modeling converter gas (BOFG) in a tunnel furnace. The validation procedure was conducted on a real test bed working on natural gas. Good conformity between numerical and experimental data was obtained.
- Simulations of converter gas combustion carried out for the current operating parameters of the tunnel furnace show promising results for nitric oxide emission. NO at the exit of the furnace was found to be more than three times lower in comparison with the NO emission from the combustion of natural gas in this furnace.
- Carbon dioxide emission was nearly three times higher from the combustion of converter gas if compared to natural gas. However, one should note that converter gas is a by-product from steel manufacturing and due to its high CO content (over 60%) it cannot be released into the ambient air. Therefore, converter gas is combusted and CO_2_ is formed anyway. The proposed idea was to utilize converter gas as substitute for of natural gas. Thus, the CO_2_ reduction comes from eliminating natural gas from fueling a tunnel furnace.
- By applying BOFG instead of natural gas for heating a tunnel furnace, it should bring significant financial savings regarding gas purchasing costs.
- Taking the above into account, BOFG, due to its high energy potential, may become an attractive fuel, even though it contains a high carbon fraction in CO and CO_2_.

## Figures and Tables

**Table 1:** Technical parameters of tunnel furnace.

Parameters	Zone No. and Furnace Length (m)					
	I 7.3	II 16	III 24	IV 32.5	V 41	VI 49
Gas flowrate, ${\dot{V}}_{NG}$, $\left(\frac{m^{3}}{s}\right)$	0.068	0.035	0.026	0.019	0.031	0.016
Air flowrate, ${\dot{V}}_{air}$, $\left(\frac{m^{3}}{s}\right)$	0.681	0.353	0.256	0.186	0.261	0.136
Temperature of steel sheets, (K)	1113	1153	1173	1193	1193	1193
Excess air ratio, λ	1.05	1.05	1.05	1.05	0.90	0.90

**Table 2:** Parameters of the BOFG powered furnace adopted for calculations.

Parameters	Zone No. and Furnace Length (m)					
	I 7.3	II 16	III 24	IV 32.5	V 41	VI 49
Gas flowrate, ${\dot{V}}_{BOFG}$, $\left(\frac{m^{3}}{s}\right)$	0.292	0.151	0.110	0.079	0.130	0.068
Air flowrate, ${\dot{V}}_{air}$, $\left(\frac{m^{3}}{s}\right)$	0.489	0.253	0.184	0.134	0.187	0.098
Excess air ratio, λ	1.05	1.05	1.05	1.05	0.90	0.90
Residence time, τ (s)	11.35	16.60	22.86	31.78	45.80	116.80

## Data Availability

Data available on request.
